# The Axial Spin of the Carotid Bifurcation

**DOI:** 10.3390/diagnostics13193122

**Published:** 2023-10-04

**Authors:** Mihaela Daniela Manta, Mugurel Constantin Rusu, Sorin Hostiuc, Alexandra Diana Vrapciu, Bogdan Adrian Manta, Adelina Maria Jianu

**Affiliations:** 1 Department of Anatomy, Faculty of Medicine, Victor Babeș University of Medicine and Pharmacy, 300041 Timișoara, Romania; monea.mihaela@umft.ro (M.D.M.); adelina.jianu@umft.ro (A.M.J.); 2 Division of Anatomy, Faculty of Dentistry, Carol Davila University of Medicine and Pharmacy, 050474 Bucharest, Romania; alexandra.vrapciu@umfcd.ro; 3 Division of Legal Medicine and Bioethics, Faculty of Dentistry, Carol Davila University of Medicine and Pharmacy, 050474 Bucharest, Romania; sorin.hostiuc@umfcd.ro; 4 Division of Clinical Practical Skills, Faculty of Medicine, Victor Babeș University of Medicine and Pharmacy, 300041 Timișoara, Romania; manta.bogdan@umft.ro

**Keywords:** carotid artery, anatomical variation, twisted carotid, lateralized external carotid artery

## Abstract

(1) Background: Twisted carotid bifurcations (CBs) lead to lateralized external carotid arteries (ECAs). Such variants are usually reported on a case-by-case basis. We aimed to study the anatomical possibilities of the axial spin of CB. (2) Methods: Determinations were made bilaterally on a retrospectively assessed sample of 150 cases, 88 males and 62 females. The following types of the axial spin of the CB were determined: type CK1–CB in the coronal plane, with ICA lateral of ECA; type CK3–CB in the coronal plane, with ECA lateral of ICA; the oblique type OK1, with the ECA antero-medial of ICA; the oblique type OK3a, with the ICA antero-medially; the oblique type OK3b, with the ICA postero-laterally; the sagittal type SK2a, with ECA anterior of ICA. (3) Results: In the overall group of 300 CBs, type OK1 was found in 40%, type OK3a in 1%, type OK3b in 2%, type CK1 in 9%, type CK3 in 5.67%, and type SK2a in 42.33% of the bilateral BC group. The types SK2a (46.67%) and OK1 (33.33%) prevailed on the right side. The types OK1 (46.67%) and SK2a (38%) prevailed on the left side. There was no statistically significant association between gender and left or right subtypes. A very strong symmetry existed between the left and right sides (Pearson Chi2 = 53.93 *p* < 0.001) for types OK1 and SK2a. Asymmetrical types were found in different bilateral combinations. (4) Conclusions: The spin of the CB is relatively symmetrical bilaterally, especially for the variants with the ECA antero-medial or anterior to ICA.

## 1. Introduction

The common carotid artery (CCA) bifurcates typically at the height of the upper border of the thyroid cartilage into the external (ECA) and internal (ICA) carotid arteries [1]. According to descriptions in classical anatomy textbooks, the initial part of the ECA is placed medial and anterior to the ICA, but at the gonial level it crosses the ICA and externally reaches it [2]. Aberrant anatomy is defined as any significant variation from the usual relationship of the ECA [3]. However, the idea of an anatomical norm is paradoxical as the boundaries between what is considered normal and what is anomalous are blurred [4]. Medical school graduates are unfamiliar with anatomical variations, which can ultimately cause ineptitude in analyzing a surgical dissection plane or reading medical imaging data [5]. In different instances, twisted carotid bifurcations (CBs) were found. During bleeding from terminal branches of ECA, occasionally, it is necessary to consider its ligature, and it is essential to ensure that the artery being ligated is indeed the ECA rather than the ICA, as the accidental ligature of ICA could lead to hemiparesis [6]. Kamide defined three anatomical variabilities in the relative placement of ECA and ICA: type 1, in which and the ECA runs medially; type 2, when the ICA and ECA run to overlap sagittally; and type 3, when the ICA runs medially and the ECA runs laterally [7]. Surprisingly, little attention has been paid to studying the different variabilities of CB twisting, and thus we aimed to study these using computed tomography angiograms based on Kamide types.

## 2. Materials and Methods

There were used 153 archived angioCT files. Inclusion criteria: good quality of scans; adequate vertical height; and no pathological processes distorting the carotid anatomy. Exclusion criteria: scans inadequate for observing the carotid arteries; pathological processes nearing the carotid arteries and distorting their anatomical features; and previous surgery in the neck, hyperextension, or excessive lateral rotation of the neck during the procedure. Being a retrospective study on archived files, informed consent was waived. The research followed principles from The Code of Ethics of the World Medical Association (Declaration of Helsinki). The responsible authorities (affiliation 1) approved the study (approval no. 45/4 September 2020).

The CTAs were performed using a 32-slice scanner (Siemens Multislice Perspective Scanner, Forcheim, Germany), with a 0.6 mm collimation, 0.75 mm thick reconstruction, and a 50% overlap for a multiplanar maximum intensity projection and three-dimensional volume rendering technique, as previously described [8]. The cases were documented using Horos for iOS (Horos Project), as in previous studies [9]. Findings were verified on two-dimensional planar reconstructions and were documented with three-dimensional volume renderings.

**Figure 1:**
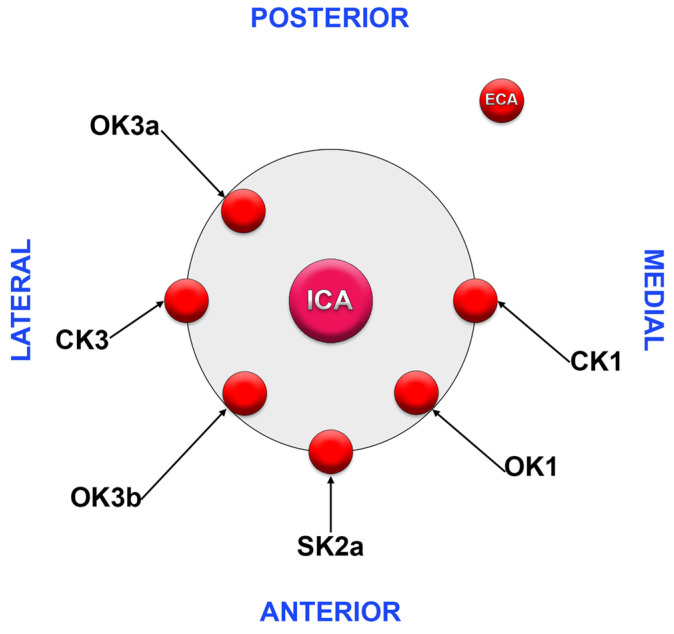
Types of axial spin of the carotid bifurcation. Diagram: horizontal section, right side, superior view.

We assessed the axial rotation of the CB in sagittal (S), coronal (C), and oblique (O) planes, respectively. We correlated these types with Kamide types (see Introduction). This resulted in the documentation of the following types: CK1 (CB in the coronal plane with ICA laterally and ECA medially), CK3 (CB in the coronal plane with ECA laterally and ICA medially), OK1 (obliquely oriented CB with ECA antero-medially and ICA postero-laterally, normal anatomical variant), OK3a (obliquely oriented CB with antero-medial ICA), OK3b (obliquely oriented CB with postero-medial ICA), and SK2a (sagittally oriented CB with ECA anterior of ICA) (Figure 1).

Statistical analysis was performed using SPSS v.29 for MacOS. We used the Pearson Chi2 test to assess significant associations between qualitative variables. A *p*-value below 0.05 was considered statistically significant.

## 3. Results

We excluded three cases. Determinations were thus made bilaterally on a retrospectively assessed sample of 150 cases: 88 males and 62 females (sex ratio = 1.4).

**Figure 2:**
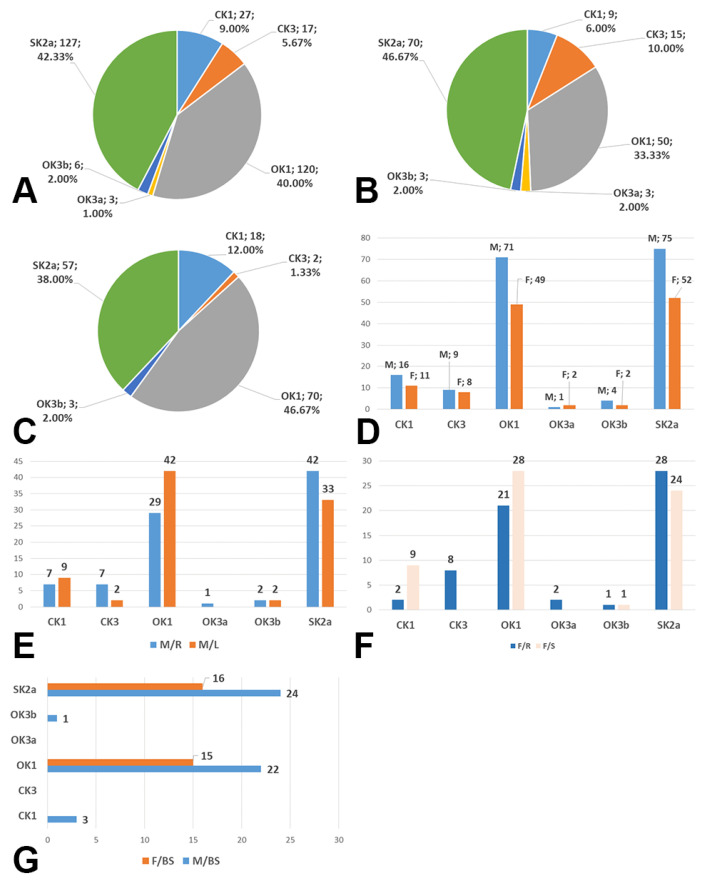
(**A**) Prevalence of carotid bifurcation axial rotation types combined with Kamide types in the bilaterally investigated group (*n* = 300). (**B**) Prevalence of carotid bifurcation axial rotation types combined with Kamide types in the right sides of the investigated group (*n* = 150). (**C**) Prevalence of carotid bifurcation axial rotation types combined with Kamide types in the left sides of the investigated group (*n* = 150). (**D**) Gender distribution (M: male, F: female) of carotid bifurcation axial rotation types (number of cases), bilaterally. (**E**) Types of carotid bifurcation axial rotation in men (M) right (R) and left (L) sides. (**F**) Types of carotid bifurcation axial rotation in women (F) right (R) and left (L) sides. (**G**) Types of axial rotation with bilateral symmetry by sex (M—male, F—female). BS: bilateral symmetry.

In the overall group (*n* = 150, 300 CBs), type OK1 was found in 40% of BCs, type OK3a in 1%, type OK3b in 2%, type CK1 in 9%, type CK3 in 5.67% and type SK2a in 42.33% of the bilateral BC group (Figure 2A). We did not identify the sagittal type of CB with the ICA anterior of ECA.

On the right side, in the overall group (*n* = 150, 150 CBs), type OK1 was identified in 33.33% of BC, type OK3a in 2%, type OK3b in 2%, type SK2a in 46.67%, type CK1 in 6%, and type CK3 in 10% (Figure 2B).

On the left side in the overall group (*n* = 150, 150 CBs), we did not identify type OK3a; type OK1 was identified in 46.67% of CBs, type OK3 in 2%, type SK2a in 38%, type CK1 in 12%, and type CK3 in 1.33% (Figure 2C).

In males (NM = 88), bilaterally (*n* = 172 CBs), we found 16 CBs with type CK1, 9 CBs with type CK 3, 71 CBs with type OK1, 1 CB with type OK3a, 4 CBs with type OK3b, and 75 CBs with type SK2a (Figure 2D). In females (NF = 62), bilaterally (*n* = 124), type CK1 was detected in 11 CBs, type CK3 in 8 CBs, type OK1 in 49 CBs, type OK3a in 2 CBs, type OK3b in 2 CBs, and type SK2a in 52 CBs (Figure 2D).

When bilaterally comparing the right and left sides, the identified CB types resulted in the following distribution in males: type CK1—7/9, type CK3—7/2, type OK1—29/42, type OK3a—1/0, type OK3b—2/2, and type SK2a—42/33 (Figure 2E).

When bilaterally comparing the right and left sides, the identified CB types resulted in the following distribution in women: type CK1—2/9, type CK3—9/0, type OK1—21/28, type OK3a—2/0, type OK3b—1/1, and type SK2a—28/24 (Figure 2F).

In the 150 cases, 54% showed a bilateral symmetry of CB axial rotation: 50/88 cases in males showed the bilateral symmetry of CB axial rotation, and 31/62 cases in females had bilateral symmetry of this morphological variable. Their distribution by type is shown in Figure 2G.

**Figure 3:**
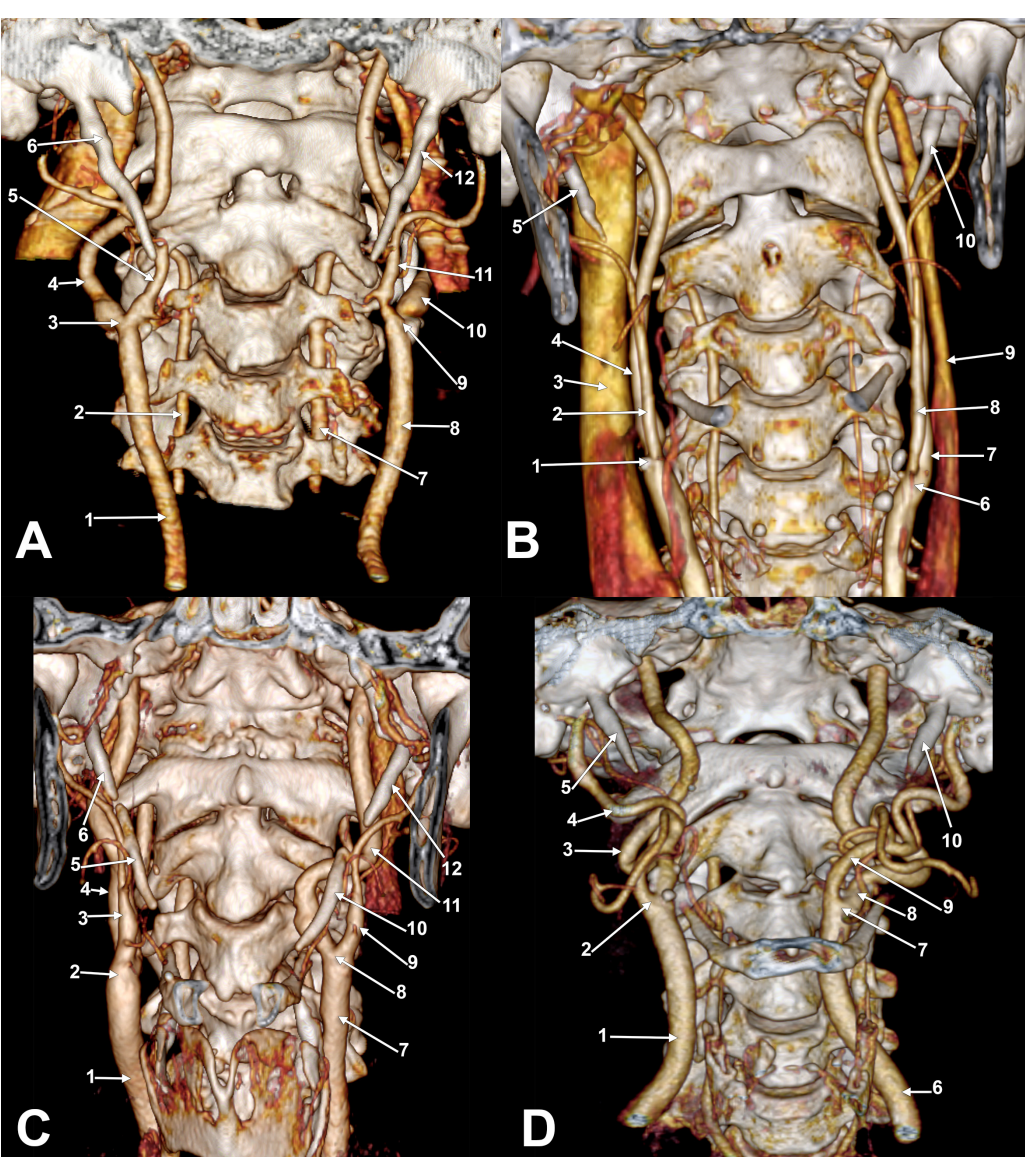
(**A**) Bilateral asymmetrical axial rotation of carotid bifurcation (bilateral OK1 type). Three-dimensional volume rendering. Anterior view. Right side: 1. common carotid a.; 2. vertebral a.; 3. carotid bifurcation; 4. internal carotid a.; 5. external carotid a.; 6. styloid process. Left side: 7. vertebral a.; 8. common carotid a.; 9. carotid bifurcation; 10. internal carotid a.; 11. external carotid a.; 12. styloid process. (**B**) Bilateral asymmetrical axial rotation of carotid bifurcation (bilateral SK2a type). Three-dimensional volume rendering. Anterior view. Right side: 1. carotid bifurcation; 2. external carotid a.; 3. internal jugular v.; 4. internal carotid a.; 5. styloid process. Left side: 6. carotid bifurcation; 7. internal carotid a.; 8. external carotid a.; 9. internal jugular v.; 10. styloid process. (**C**) Bilateral asymmetrical axial rotation of carotid bifurcation (right SK2a and left CK3 types). Left carotid helix. Three-dimensional volume rendering. Anterior view. Right side: 1. common carotid a.; 2. carotid bifurcation; 3. external carotid a.; 4. internal carotid a.; 5. ossified stylohyoid lig.; 6. styloid process. Left side: 7. common carotid a.; 8. carotid bifurcation; 9. external carotid a.; 10. ossified stylohyoid lig.; 11. internal carotid a.; 12. styloid process. (**D**) Bilateral asymmetrical axial rotation of carotid bifurcation (right CK3 and left CK1 types). Right carotid helix. Three-dimensional volume rendering. Anterior view. Right side: 1. common carotid a.; 2. carotid bifurcation; 3. internal carotid a.; 4. external carotid a.; 5. styloid process. Left side: 6. common carotid a.; 7. carotid bifurcation; 8. internal carotid a.; 9. external carotid a.; 10. styloid process.

**Figure 4:**
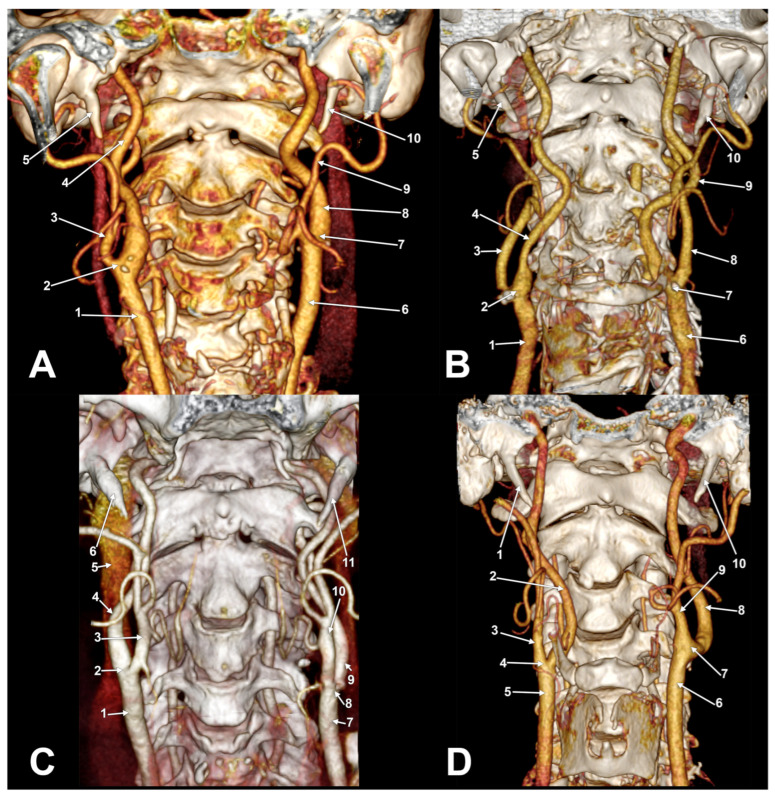
(**A**) Bilateral asymmetrical axial rotation of carotid bifurcation (right CK3 and left OK1 types). Three-dimensional volume rendering. Anterior view. Right side: 1. common carotid a.; 2. carotid bifurcation; 3. external carotid a.; 4. internal carotid a.; 5. styloid process. Left side: 6. common carotid a.; 7. carotid bifurcation; 8. internal carotid a.; 9. external carotid a.; 10. styloid process. (**B**) Bilateral asymmetrical axial rotation of carotid bifurcation (right OK3a and left CK3 types). Left carotid helix. Three-dimensional volume rendering. Anterior view. Right side: 1. common carotid a.; 2. carotid bifurcation; 3. external carotid a.; 4. internal carotid a.; 5. styloid process. Left side: 6. common carotid a.; 7. carotid bifurcation; 8. external carotid a.; 9. internal carotid a.; 10. styloid process. (**C**) Bilateral asymmetric carotid bifurcation axial rotation (right OK1 and left SK2a types). Three-dimensional volume rendering. Anterior view. Right side: 1. common carotid a.; 2. carotid bifurcation; 3. external carotid a.; 4. internal carotid a.; 5. internal jugular v.; 6. styloid process. Left side: 7. common carotid a.; 8. carotid bifurcation; 9. internal carotid a.; 10. external carotid a.; 11. styloid process. (**D**) Bilateral asymmetrical axial rotation of carotid bifurcation (right CK3 and left OK1 types). Three-dimensional volume rendering. Anterior view. Right side: 1. styloid process; 2. internal carotid a.; 3. external carotid a.; 4. carotid bifurcation; 5. common carotid a. Left side: 6. common carotid a.; 7. carotid bifurcation; 8. internal carotid a.; 9. external carotid a.; 10. styloid process.

**Figure 5:**
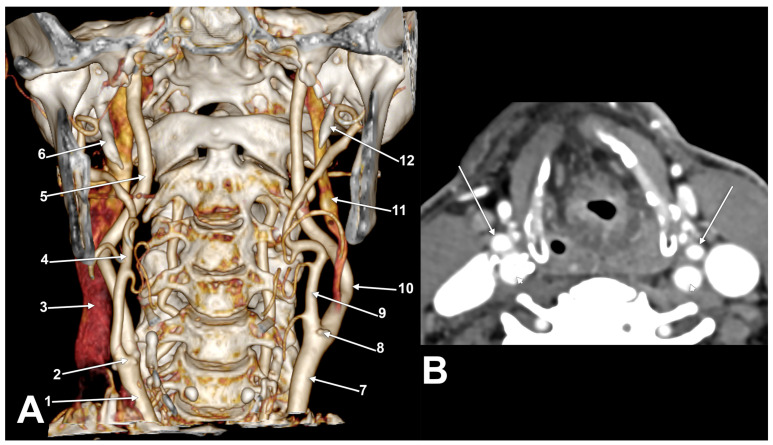
(**A**) Bilateral asymmetrical axial rotation of carotid bifurcation (right OK3b and left OK1 types). Right carotid helix. Three-dimensional volume rendering. Anterior view. Right side: 1. common carotid a.; 2. carotid bifurcation; 3. internal jugular v.; 4. external carotid a.; 5. internal carotid a.; 6. styloid process. Left side: 7. common carotid a.; 8. carotid bifurcation; 9. external carotid a.; 10. internal carotid a.; 11. internal jugular vein; 12. styloid process. (**B**) Bilateral OK3b type of axial rotation of the carotid bifurcation. Axial slice. External carotid arteries (arrows) and internal carotid arteries (arrowheads) are shown.

There was no statistically significant association between gender and left or right side subtypes (Pearson Chi2 = 4.82, *p* = 0.30 for the right side, and Pearson Chi2 = 0.887, *p* = 0.926 for the left side. There is a very strong symmetry between the left and right sides (Pearson Chi2 = 53.93, *p* < 0.001), especially for types OK1 (Figure 3A) and SK2a (Figure 3B). Asymmetrical types were found in different bilateral combinations (Figure 3C,D, Figure 4 and Figure 5A). One male case was found with the bilaterally symmetrical type OK3b (Figure 5B), and three others with bilaterally symmetrical type CK1.

## 4. Discussion

The usual carotid lesion lies at the CB [10]. However, the anatomy of the CB is highly variable concerning its vertical position [11,12,13,14,15], its relationship with the hyoid bone [16], and its axial spin. In previous studies, the vertical position of the CB was referred to vertebral landmarks, anterior cervical landmarks, or both [17,18,19,20,21,22,23,24,25,26]. The twisted carotid artery (TCA)—or ECA and ICA transposition, or twisted CB—is a variant in which the ICA is medial to the ECA [27,28]. If the ECA is angiographically noted to arise posterior to the ICA or to be wholly superimposed upon the ICA in the lateral projection, the presence of a twisted CB is suggested [29]. Ueda et al. noted that the first description of a lateralized ECA belongs to Hyrtl in 1841 [14]. As Honda et al. observed, specific studies of twisted CBs are rare [27].

According to different authors, the twisted CB occurs more frequently on the right side [29,30,31]. It is also significantly more common in older patients, female patients, and patients with stenosis or occlusion of the ICA at the level of the CB [32]. Katano and Yamada observed that carotid torsion is clockwise [33]. Various studies reported a significant incidence of twisted CBs (between 3.6% and 19.5%) [22,28,32,33,34,35]. The twisted CB incidence was found to be 5.35% by Prendes et al. (1980) and Ito et al. (2016), but in different-sized samples [3,31]. In the present study, a lateral ECA corresponds to types CK3, OK3a, and OK3b, found to be 5.67%, 1%, and 2%, respectively. This resulted in an overall prevalence of lateralized ECA of 8.67% in our sample. The different incidences of the twisted CB could be due to the absence of strict criteria for defining the degree of axial rotation (spin) of the CB and considering the ECA as lateral to the ICA; the definition of a lateralized ECA is operator-dependent and subject to biases, and the actual incidence remains elusive [28]. In this regard, using different classification systems, such as Kamide types in this study, could better evaluate the incidence of the twisted CBs. The phenomenon of migration of the carotid arteries in reference to the pharynx should not be ignored [36]. Thus, a variable axial spin of the CB could occur at different times in the same patient. Therefore, a preoperative assessment of the carotid anatomy could be considered even though the patient was scanned previously.

Numerous reports of lateralized ECAs could not indicate the specific incidence of this variant [14,30,34,37,38,39,40,41,42,43]. A few other studies simply observed the lateral position of ECA referred to the ICA and did not consider specific types of the axial spin of CB [3,29,31,32,33,35,44]. In these studies, lots of different sizes and different methods were used. Various surgical techniques for arterial ligatures in the carotid triangle are based on the usual disposition of the ECA and ICA [37]. The standard anatomical disposition could be modified either by a twisted CB variant or by different tortuosities of carotid arteries [45,46,47], but not exclusively.

As in this study, few other research groups determined distinctive types in the relationship between ECA and ICA [2,7,48]. Uno et al. (2020) used the three types previously defined by Kamide et al. (2016). In Kamide’s angiographic study, the sagittal type 2 prevailed (51.7%), followed by type 1 (41.4%) and type 3 (6.9%), while in Uno’s 3D computed tomographic angiography study prevailed the type 1, with the ECA medially (64.4%), followed by the types 2 (27.8%) and 3 (7.8%) [7,48]. The K1 variants (CK1 and OK1) we found here occurred in 49% of CBs, the SK2a variant occurred in 42.33%, and the K3 variants (CK3, OK3a, and OK3b) incidence was 8.67%.

Delic et al. (2009) evaluated 50 MRI scans and grouped the anatomical variabilities of ECA-ICA inter-relationship into four types: type 1, with the ECA medially and ascending anterior to the ICA (90%); type 2, with the ECA lateral to the ICA (7%; in 2% this type had bilateral symmetry); type 3, with divergent position, in which the ICA (medially) and ECA (laterally) move away from each other without intersecting (1%); and type 4, in which the ICA and ECA intersect twice (1%) [2], resulting in a bicarotid helix, similar to what we found in our study. The crossing height is symmetrical in 18% of cases [2]. However, all Delic’s types 2–4 indicate lateralized ECAs. These were found in 10% of cases. These findings suggest that although the ECA is lateralized, its further course could parallel the ICA, diverge from it, or cross it twice. Since anatomical textbooks often describe the ECA and ICA crossing only once, a bicarotid helix is rather unexpected and could determine wrong surgical gestures if this possible variant remains unknown.

In 300 CBs, we found just 40% of the standard OK1 variant of CB. Accordingly, the aberrant variants of the axial spin of the CB prevail over the normal ones. Interestingly, we found strong symmetry between the left and right sides, especially for types OK1 and SK2a. These types are antero-medial with the ECA and anterior to the ICA, respectively, and thus can be regarded as normal. Therefore, normal variants of the CB axial spin are relatively bilaterally symmetrical.

The possibility of a twisted CB must be considered when performing arterial ligatures in the carotid triangle to avoid damage to the ICA or hemorrhagic accidents [2]. Changes in the anatomy of the CB are significant during surgery, as such modifications alter the expected relationships with different nerves, such as the hypoglossal nerve, vagus nerve, or superior laryngeal nerve [28,30]. The internal branch of the superior laryngeal nerve could cross between a lateralized ECA and the ICA, being exposed at risk during surgery [34]. It was found that during carotid endarterectomy, there is a statistically significant incidence of dysfunction in the external branch of the superior laryngeal nerve [28]. This may not be a rule, as the clamps could be placed in a way that rotates the carotid axis to facilitate endarterectomy, and, at the end of the procedure, unclamping the carotid axis would allow the CB to return to its native anatomical position.

As previously observed, there are significant interindividual differences in CB anatomy and within individuals [49]. The size of the carotid arteries has a certain functional role [49], while the arterial geometry seems rather surgically important. Not only the axial spin of the CB is variable, but also the angle of the CB [50,51]. The latter is seemingly age dependent [50]. Moreover, in cases with twisted CB, when the ECA is lateral to the ICA, the anterior branches of the ECA, i.e., the superior thyroid, lingual, and facial arteries, cross over the ICA to continue with a typical course [37]. A linguofacial trunk emerging from a lateralized ECA was also reported [39]. All these arteries should be ligatured during surgery [28]. Nevertheless, a twisted CB is clinically meaningful during carotid endarterectomies [30]. However, after correcting the anatomical location of a twisted CB, surgical duration and adverse event rates do not significantly differ between patients with and without such a twisted CB [48].

Twisted CBs could be preferentially found in the right severely atherosclerotic carotids in cases with diabetes mellitus and/or hypertension [33]. Carotid endarterectomy of twisted carotids can be safely accomplished, sometimes with correction of the carotid position [33]. Circumferential dissection and medial mobilization of the ECA provide suitable exposure for carotid endarterectomy [34]. MDCT angiography or 3D-DSA is recommended in twisted carotid cases to add information on steric relationships [33]. A case-by-case 3D evaluation should refer to particular anatomical possibilities of the axial spin of the CB, as we determined here. Cross-sectional imaging is critical in identifying abnormalities and planning surgical and nonsurgical therapies [52].

## 5. Conclusions

The spin of the CB is relatively bilaterally symmetrical, especially for the variants with the ECA antero-medial or anterior to ICA. The axial spin of the CB should be evaluated preoperatively on a case-by-case basis, and it also should be used to improve primary anatomical teaching.

## Data Availability

No new data were created or analyzed in this study. Data sharing does not apply to this article.
